# “That’s kind of like the big struggle right now is can we get PrEP?”: Facilitators and Barriers to PrEP Uptake Among Active Duty Gay and Bisexual Men

**DOI:** 10.1007/s13178-021-00622-6

**Published:** 2021-08-20

**Authors:** Raiza M. Beltran, Ashley C. Schuyler, Cherie S. Blair, Jeremy T. Goldbach, Carl A. Castro, Ian W. Holloway

**Affiliations:** 1 Division of Infectious Diseases, UCLA David Geffen School of Medicine, University of California Los Angeles, 10833 Le Conte Avenue, Los Angeles, CA USA; 2 School of Social and Behavioral Health Sciences, College of Public Health and Human Sciences, Oregon State University, Corvallis, OR USA; 3 Suzanne Dworak-Peck School of Social Work, University of Southern California, Los Angeles, CA USA; 4 Department of Social Welfare, School of Public Affairs, UCLA Luskin, University of California, Los Angeles, Los Angeles, CA USA

**Keywords:** GBM, Active duty military, HIV and military healthcare system, PrEP access and uptake

## Abstract

**Introduction:**

The US Military is experiencing a rise in HIV infections among gay and bisexual men (GBM) serving on active duty, yet little is known about this population’s uptake of pre-exposure prophylaxis (PrEP), an evidence-based intervention for HIV prevention. This mixed methods study examines the facilitators and barriers to PrEP access and uptake among active duty GBM.

**Methods:**

Active duty GBM were recruited using respondent-driven sampling (2017 and 2018). Participants (*n* = 93) answered quantitative survey questions on PrEP interest and accessibility. Another set of participants (*n* = 10) discussed their PrEP experiences in qualitative interviews*.* We conducted descriptive and bivariate analyses of quantitative data, while qualitative data were analyzed using structural and descriptive coding techniques.

**Results:**

Approximately 71% of active duty GBM indicated interest in accessing PrEP. A greater proportion of those who disclosed (vs. did not disclose) their sexual orientation to their military doctor discussed (*p* < 0.001) or accessed (*p* = 0.017) PrEP. The following qualitative themes emerged: (1) providers’ negative views and knowledge gaps related to PrEP; (2) lack of a systems approach to PrEP access; (3) confidentiality concerns; and (4) reliance on peer networks for PrEP guidance and support.

**Conclusions:**

Study results indicate that active duty GBM are interested in and want to discuss PrEP with their military doctors, but gaps in providers’ PrEP-related knowledge and skills, as well as mistrust in the military health care system, remain.

**Policy Implications:**

A system-wide approach that addresses confidentiality concerns and removes procedural barriers to PrEP access is recommended to improve PrEP uptake in this population.

## Introduction

Biomedical advances in the prevention of HIV, including pre-exposure prophylaxis (PrEP), have helped lower HIV infection rates in the US. As recently as 2019, the US Preventive Task Force (USPTF) assigned a grade “A” recommendation, indicating that all persons at high risk for HIV acquisition be considered for PrEP, including sexually active men who have sex with men (MSM) (Centers for Disease Control & Prevention, 2018; Walensky & Paltiel, 2019). Given that PrEP reduces HIV acquisition risk by 96 to 100% (Anderson et al., 2012; Grant et al., 2014), it is imperative to examine its potential usefulness within the US military, which has experienced rising HIV incidence, particularly among Black gay and bisexual men (GBM) service members (Blaylock et al., 2018b; Hakre et al., 2011). In fact, approximately 350 new HIV infections are detected among military personnel each year, as the Armed Forces Health Surveillance Branch find increased demand for HIV preventive services by military members and their beneficiaries (Blaylock et al., 2018b; Okulicz et al., 2017). Yet few studies have been conducted to understand facilitators and barriers in PrEP access and uptake among active duty GBM (Blaylock et al., 2018a, b).

### The Legacy of Don’t Ask Don’t Tell (DADT)

Limited knowledge on the prevention and acquisition of HIV among military service members can be partially attributed to the 1994 implementation and eventual 2010 repeal of the Department of Defense (DoD) directive known as “Don’t Ask and Don’t Tell” (DADT) (Hoover et al., 2017; Okulicz et al., 2017). This policy prohibited nearly 71,000 lesbian, gay, and bisexual (LGB) persons from openly serving in the military, thereby limiting their ability to discuss their sexual health history with medical providers within the military healthcare system (Biddix et al., 2013; Gates, 2010; Hakre et al., 2012; Rerucha et al., 2018). Therefore, it is not surprising that during the DADT era, a quarter of LGB-identified service members sought care in civilian health facilities for their sexually transmitted infections (STIs) due to fear of reprisal by the military because of their sexual orientation (Biddix et al., 2013; Hakre et al., 2012; Smith, 2008). DADT was implemented to prevent discrimination and harassment of LGB service members but instead caused direct and indirect harm to the population it was intended to protect by prompting mistrust among fellow military personnel and of military health providers (Biddix et al., 2013; McNamara et al., 2020), which produced stressors that contributed to poorer physical and mental health outcomes among LGB service members (Holloway et al., 2020; McNamara et al., 2020; Ramirez & Sterzing, 2017) and reduced researchers’ ability to gain accurate information about this population (Gates, 2010; Hoover et al., 2017). 

### HIV Prevention Among Active Duty GBM

National estimates from 2017 indicate that approximately 4% of men serving in the military identified as gay or bisexual (Hoover et al., 2017). With the repeal of DADT, renewed efforts are underway to focus on HIV prevention in this population, which is disproportionately affected by the HIV epidemic (Blaylock et al., 2018b; Hakre et al., 2012; Okulicz et al., 2017). Among the first post-DADT studies which sought input directly from active-duty military personnel was an online pilot survey among newly diagnosed HIV-positive sailors and Marines, with findings pointing to condomless sex and having concurrent sexual partners as the most common risk factors for HIV transmission (Hakre et al., 2012). Additional studies examing the medical records of HIV-positive military personnel indicated that an HIV-positive diagnosis was associated with the following individual characteristics: Black race, single relationship status, male gender, recent STI diagnosis, history of mental illness, and being stationed in the Southern US (Hakre et al., 2011, 2017).

To date, only three studies have been published examining PrEP use among military service members, two of which were authored by Blaylock and his colleagues in 2018 (Blaylock et al., 2018a, b). In both studies, Baylock et al. (2018a, b) reviewed medical records of GBM service members from different study sites, who were prescribed PrEP between 2013 to 2016; results demonstrated that PrEP uptake was associated with having condomless sex, sexual contact with multiple male partners, and exposure to sexual partners with a known HIV diagnosis. For both studies, PrEP was prescribed to predominately white (47–55%), young (40–58%), and male (98–99%) military personnel (Blaylock et al., 2018a, b). Only about 19% of Black service members received a PrEP prescription in both of the studies (Blaylock et al., 2018a, b). Finally, in surveying GBM military personnel who subscribed to a closed social media group, a recent study found that PrEP daily tablet, long acting implants and injections were the desired uptake methods in this population (Gutierrez et al., 2021). Recognizing the need to reduce HIV transmission among US military servicemembers, the US Department of Defense issued a memorandum in 2018 requiring military health facilities to offer a “pathway” to access PrEP (US Department of Defense Agency, 2018), yet little is known about the policy’s implementation.

It is estimated that nearly 12,000 GBM military service members may be eligible for PrEP (Blaylock et al., 2018b). There are currently no published qualitative studies examining this population’s perceptions of or intentions to use PrEP, and only three quantitative studies were conducted, two of which used a review of patients’ medical records (Blaylock, et al., 2018a, b). Using a mixed-methods approach, this study is among the first to directly assess self-reported PrEP interest and accessibility, as well as the perceived facilitators and barriers to PrEP uptake. Specifically, this study sought to answer the following research questions: 1) What is the interest in and access to PrEP among active duty military GBM? and 2) What are the perceived barriers and facilitators to PrEP uptake among active duty military GBM?

## Methods

Data for this analysis are from the Military Acceptance Project (MAP), a two-phase mixed-methods study conducted to better understand the integration, acceptance and well-being of lesbian, gay, bisexual and transgender (LGBT) service members in the US military. Briefly, MAP involved a formative qualitative phase consisting of individual interviews with a small sample of active duty LGBT service members, which informed the development of an anonymous online survey for quantitative data collection among a larger sample of LGBT and non-LGBT service members. The qualitative and quantitative phases included independent samples.

### Qualitative Phase

#### Recruitment

Qualitative participants were recruited by key informants of the study’s Expert Advisory Panel (EAP), comprised of former and current LGBT military personnel. Specifically, EAP members distributed flyers with study information to eligible military personnel in their networks. Service members who were interested in participating contacted the study team by phone or email to be screened for eligibility and schedule an interview. A $25 incentive was offered to participants.

#### Procedure and Instruments

Individual interviews, lasting approximately 90 min, were conducted with 42 LGBT military service members, 22 of whom identified as GBM, from the four main branches of the US military (Air Force, Army, Marines, Navy). Following a semi-structured life history calendar (LHC) protocol (Freedman et al., 1988), participants were asked about their physical and psychological health, life stressors, and social support networks during their military service. Although participants were not asked about PrEP specifically, several GBM interviewees (*n* = 10) spontaneously described their perceptions of or experiences with PrEP as part of larger discussions about sexual health and military healthcare.

#### Analysis

We used structural and descriptive coding techniques (Saldaña, 2021) to analyze the qualitative data describing GBM participants' PrEP-related beliefs and experiences. Three co-authors (RB, AS, IH) reviewed passages from GBM participants who voluntarily discussed PrEP (*n* = 10) to develop a preliminary codebook reflecting men’s perceptions of and experiences with PrEP. Two co-authors (RB, AS) coded all transcripts and worked collaboratively to identify major themes related to PrEP access barriers and facilitators.

### Quantitative Phase

#### Recruitment

Respondent driven sampling (RDS), found to be effective in recruitment of hard-to-reach populations, was employed for the quantitative phase of MAP (Heckathorn, 1997). RDS relies on select participants, also known as “seeds,” from varied ideal stratum to recruit members within their social networks, with the expectation that the referral chain will grow, thereby increasing the sample size of the study (Heckathorn, 1997). RDS seeds for the quantitative phase included 1) military contacts of study team members; 2) referrals from the study’s EAP; and 3) participants from the study’s initial qualitative phase who were willing to recruit members of their networks. To reach LGBT service members who may not have been “out” (i.e., disclosed their sexual orientation/gender identity), we supplemented RDS recruitment with targeted advertisements in each military branch’s official digital and print newspaper, as well as private military Facebook groups known to study team members. Participants received a $25 e-gift card for completing the survey as well as $10 for each eligible participant they helped recruit into the study.

#### Procedures and Instruments

A total of 93 respondents answered the questions pertaining to PrEP interest and accessibility, and therefore comprised the analytic sample for quantitative analyses. After being provided a description of PrEP, interest in the medication was assessed by asking: “Are you interested in having access to PrEP?” with a binary response option provided of “yes” or “no”. Those who answered affirmatively were asked two additional questions providing a binary response option of “yes” or “no”: 1) “Have you ever talked to a military doctor about PrEP?” and 2) “Do you have access to PrEP though a military healthcare provider?” Additionally, questions regarding respondents’ health care utilization, such as if the respondent accessed healthcare in the last year, from either a military or civilian provider, if the participant’s sexual orientation was disclosed during the healthcare visit, as well as their social and military demographics, were posed in the questionnaire.

#### Analysis

Descriptive statistics (frequency, percentage) were obtained using the sample, stratifying based on response to each of the 3 questions that pertained to PrEP. Bivariate analyses were conducted using chi-square and Fisher’s exact tests, where appropriate, for categorical variables and Kruskal–Wallis tests for continuous variables.

## Results

### Qualitative Phase

Qualitative findings were derived from interviews with 10 male service members (age 22–37 yrs., mean age = 28.6 yrs.; all identifying as White race) identifying as gay (*n* = 9) or bisexual (*n* = 1), and serving in the Navy (*n* = 6), Air Force (*n* = 3), and Army (*n* = 1). Participants discussed their perceptions and experiences related to PrEP. It is important to note that participants were not prompted about PrEP during their interview, but discussed it extemporaneously as part of larger discussions about sexual health and military healthcare. Two participants mentioned their occupation as military healthcare providers; we note this in the presentation of our findings to help contextualize data from the perspective of service members working in the military healthcare system. Major themes that emerged related to barriers and facilitators of PrEP accessibility were 1) providers’ knowledge gaps and/or negative beliefs related to PrEP; 2) the lack of a systems approach to PrEP access; 3) confidentiality concerns; and 4) reliance on peer networks for PrEP information and support.

#### Providers’ Knowledge Gaps and Beliefs About PrEP

Study participants (*n* = 7) described difficulty in seeking PrEP within the military healthcare system due to providers’ PrEP knowledge gaps, or negative beliefs about PrEP and/or GBM’s sexual behavior. Among these participants, several (*n* = 4) discussed encountering military healthcare providers with little and/or incomplete knowledge about PrEP, often prompting service members to either seek out multiple providers or educate providers about PrEP themselves.

*I went to my flight doc...and asked about it [PrEP]... like, he had never heard of it and had no experience with the medicine. And then, another doctor had never experienced it...But, the doctor that I had actually was a good doc and he referred me to San Antonio to the medical hospital there and they actually have an infectious disease department that handles...Truvada.* – P10 (gay male, Navy)

*I started PrEP...through the military a number of years ago. I first had to go through my primary physician in the military and ask them if they could either refer me or prescribe this medication. He had absolutely no idea what this was. He had no idea it existed. He had no clue that it was recommended for certain groups, specifically LGBT groups who are at risk for acquiring HIV. He had no clue what to do. Luckily, being a doctor, I can tell him what to do and I told him the referrals that I needed to make this happen.* – P6 (gay male, Navy, military healthcare provider)

Although participants P6 and P10 were able to successfully obtain PrEP, it required taking the initiative to seek out additional providers or to help providers understand how to facilitate PrEP access. One participant indicated that providers’ gaps in PrEP knowledge fostered a lack of confidence in military healthcare.

*They [doctors] have no idea what it [PrEP] is and they have to Google it...It’s upsetting and it makes me...I have less confidence in the military healthcare providers over this... not only do they not understand, so then trying to get them to advocate for me, or even to convince them that I’m a candidate for this drug, is extremely difficult*. – P7 (bisexual male, Air Force)

As suggested by these participants, the gap in providers’ PrEP knowledge can act as a barrier to PrEP access. Additionally, some participants (*n* = 3) described perceptions or vicarious experiences of encountering negative beliefs about PrEP and/or about their sexual behavior in seeking the medication from military healthcare providers. Service members who inquired about PrEP faced stigmatizing attitudes, biases, and assumptions from providers.

*I know that some of my friends out here, some of them, they went to talk about...PrEP drug with their healthcare provider and one of them was like “no, I won’t put you on that. I won’t... you’re not supposed to be having that many sexual partners”...His provider said “no, I can’t endorse...because I feel like that’s unhealthy by you going to damage your health by being on it.* – P9 (gay male, Navy)

*I know a friend who went in and asked about it, about PrEP, specifically and the nurse was basically stonewalling about it...this nurse basically just said...you can either expose yourself to the dangers of HIV or you can go date girls. It’s your choice.* – P1 (gay male, Air Force)

*And I’ve seen a lot of issues where somebody did ask for like an HIV test or PrEP and were basically told no. You know...“you should...no” and it was very clear that the doctors were not comfortable and weren’t prescribing it because... you know. Like, I think I remember somebody asked for an HIV test. The doctor said “why?” and they said “because I’m sexually active gay male” and they’re like “well, maybe you should not be so promiscuous.” It was pretty wrong. -* P5 (gay male, Navy)

In seeking out PrEP or related sexual healthcare, military GBM may face prejudice and bias from providers who hold negative beliefs about PrEP and/or their sexual behavior. In some instances, these beliefs may directly prevent PrEP access if providers are unwilling to provide the medication or other PrEP-related services. More broadly, discriminatory attitudes and behaviors perpetuate sexuality-related stigma within the military healthcare system, which may influence GBM’s comfort with or trust in seeking HIV-related care from military healthcare providers.

#### Lack of a System-Wide Approach to PrEP Access

A number of participants (*n* = 7) noted structural or bureaucratic challenges which hindered PrEP access (e.g., lack of guidance for providers, job-specific barriers, PrEP availability limited to specific locations) and are indicative of the lack of a systemic approach to PrEP in the military. PrEP access was described by several GBM as dependent on one’s location or finding a specific provider who is willing to prescribe it.

*I was living in San Diego...I heard that it was like one of the only places in the country where if you’re in the Navy, you can get a prescription for Truvada...I talked to the doctor in the infectious disease clinic and...I was like “why is this in San Diego and I couldn’t get is somewhere else?” and they’re like well, we want to carefully control how it’s given out because Truvada has double sided effects if you take it when you already have HIV, it can make it hard to... it’ll make you resistant to other drugs that might treat it, otherwise and it’s not a drug that’s meant to treat HIV; to treat you once you have HIV. It’s only to prevent you from getting it.* – P4 (gay male, Navy)

*And then, when I say get Truvada off base, I mean, say I would like to get a referral to a doctor who specializes in this off base and see them...I’d be able to do that easily because I’m a provider in the clinic and I would have to essentially talk to my colleagues that I consult with on a regular basis about my sexual health. So, I could do that. But, my concern is well how does that affect anybody else?* – P2 (gay male, Air Force, military mental health provider)

*...Some flyers are able to get it because their doctors know about PrEP, but the doctors won’t know that it’s not approved or they’ll forget to check...Maybe some doctor...they’ll kind of prescribe it anyway and nobody talks about it, whether or not it’s approved. But, yeah, for other gay men, the big question is whether or not they can get PrEP, even when they aren’t specifically barred from getting it and some have limited success and others can’t get it at all. That’s kind of like the big struggle right now is can we get PrEP? –* P7 (bisexual male, Air Force)

As suggested by participants, obtaining PrEP may require going to a specific location or military base, or seeking out providers off base/within the civilian healthcare system. That is, PrEP access in the military healthcare system is often perceived as fragmented or dependent on service members’ ability to navigate significant bureaucratic barriers, rather than a universal resource across the military. As a result, obtaining PrEP may rely on military GBM dedicating substantial time and potentially financial resources toward identifying their own pathway to PrEP.

Moreover, GBM service members in the Air Force also described specific restrictions that directly prevented PrEP prescriptions for pilots and air crew members.

*...In my case, being a flyer, I can’t take it [PrEP]. Normally...the approval process for any drug to go through the bureaucracy to get approved takes a year or two. PrEP has been out for more than a year or two and it’s still not approved. So, I’m kind of upset about that. And not only that, but PrEP is essentially just a cocktail of antiretrovirals that have already been used for a very long time...It should be available for me to take and there’s no good reason why it’s not*. – P8 (bisexual male, Air Force)

GBM military personnel expressed their frustration in having to navigate complex military bureaucracy to be prescribed PrEP. The lack of attention by healthcare providers in helping their patients through this process was notable in these participants’ experiences.

#### Confidentiality Concerns

One participant raised confidentiality concerns relating to PrEP receipt within the military health care system. This participant, a healthcare provider in the Navy, described challenges with privacy and confidentiality in his experience obtaining and using PrEP. He suggested that a perceived lack of confidentiality related to PrEP could both discourage service members from inquiring about PrEP and impede their confidence in the military healthcare system.

*The biggest problem has been confidentiality. Since I’ve been on PrEP, I have been passed between six infectious disease doctors in the same military treatment facility. There are only eight doctors in general there. So, I have been passed within 75% of the entire group...There’s a lot of reasons for this. There’s never been a malicious background to it. But I could see how very easily someone could get turned off with the idea that they don’t have a single doctor or a confidential doctor...Instead, they’re being passed from one doctor to the next, to the next, to the military and it appears that their medical history is just open for all to have a look at.* – P6 (gay male, Navy, military healthcare provider)

*I applied for a certain set of credentials through my work...One of these credentials looks at the medicines that you are taking. Because I am on PrEP, I was flagged through these credentials and the next thing I know, one of my bosses, who I had no idea had any clue about any of this is coming to me and saying “hey, why are you on Truvada?” So that was an eye-opening experience that my medical record is directly accessible to my boss...The military can pull my record at any time and show it to my bosses and say “hey, look, this is what’s going on with your sailor”. That’s a bit disappointing. I think that also discourages LGBT service members to really have confidence in the healthcare that they’re getting, to really confide in the healthcare system that they have available to them because there is a concern that it will have direct impact on their jobs.* – P6 (gay male, Navy, military healthcare provider)

For this participant, PrEP use conferred substantial threats to the confidentiality of his health status. Experiences such as these, as told by a military health care provider, offer insights into the inner workings of the military health care system wherein patients’ medical records, particularly information pertaining to their sexual health, may be shared with their superiors. This suggests that active duty service members who want to take PrEP must be willing to sacrifice a degree of privacy in order to obtain the medication, with potential implications for both their health and their military career.

#### Peer Networks for PrEP Support

Several participants (*n* = 5) described peer support networks utilized by LGBT service members for information or guidance related to PrEP access. Service members engaged with their peer networks through both social media and word-of-mouth.

*I would say we have a pretty tightly knit junior officers community of LGBT people... someone went and posted that on Facebook like “this is what the nurse told me [about PrEP]” everyone immediately, like it was a whole thing down the page being like these are all the avenues in which you can complain about this. Including other Navy doctors who were like that’s clearly not ok. Yeah, so my buddy filed this complaint and everything*. – P3 (gay male, Navy)

*Many of my colleagues who are also healthcare providers who either know that I’m gay or know that I have some level of experience in treating LGBT members will come to me, personally, asking either for their own assistance with starting PrEP. For instance, I just had a gay colleague come to me and say “hey, how do you start PrEP? I want it for myself.”* – P6 (gay male, Navy, military healthcare provider)

These quotes illustrate how peer networks may provide a vital resource for support and information among service members of various ranks and positions (including healthcare providers) who are seeking to access PrEP within the military healthcare system.

### Quantitative Phase

#### Demographic Characteristics

The median age of participants (*n* = 93) was 29 years old, most of whom identified as white (*n* = 71, 76.3%), were single (*n* = 56, 60.2%), and lived off base (*n* = 80, 87.0%). More than half of the sample had completed a bachelor’s degree or higher (*n* = 70, 75.2%) and were represented by the four branches of the US Military, including Army (*n* = 34, 36.6%), Air Force (*n* = 29, 31.2%), Navy (*n* = 20, 21.5%), and Marine Corps (*n* = 10, 10.8%), with approximately half indicating that they accessed healthcare in the last year (*n* = 51, 54.8%). About half of the participants visited a military health care provider (*n* = 47, 50.5%) and 20.4% (*n* = 19) visited a civilian healthcare provider in the last year. Many of the participants *(*n = 68, 74.7%) stated having disclosed their sexual orientation to their healthcare provider. See Table 1.

**Table 1:** Demographic information of GBM in study sample

Descriptive statistics of GBM in study sample (*n* = 93)	
**Age** (median; IQR; range)	29 (26–33; 20–49)
**Race/Ethnicity**	
White/Caucasian	71 (76.3%)
Black/African American	5 (5.4%)
Latinx/Hispanic	9 (9.7%)
Asian/Pacific Islander	4 (4.3%)
Multiracial/Other	4 (4.3%)
**Marital Status**	
Single	56 (60.2%)
Married/Partnered	32 (34.4%)
Divorced/Separated	5 (5.4%)
**Educational Level**	
High School	6 (6.5%)
Associates/Some College	17 (18.3%)
Bachelor’s	35 (37.6%)
Graduate School	35 (37.6%)
**Military Branch**	
Air Force	29 (31.2%)
Army	34 (36.6%)
Marine Corps	10 (10.8%)
Navy	20 (21.5%)
**Post**	
On-Base	12 (13.0%)
Off-Base	80 (87.0%)
**Accessed healthcare in past year**	
Yes	51 (54.8%)
**Accessed healthcare from military provider in past year**	
Yes	47 (50.5%)
**Accessed healthcare from civilian provider in past year**	
Yes	19 (20.4%)
**Disclosed sexual orientation to healthcare provider**	
Yes	68 (74.7%)

#### PrEP Interest

Approximately 71% (*n* = 66/93) of active duty GBM service members indicated that they were interested in accessing PrEP. The only statistically significant demographic difference found between respondents who were and were not interested in PrEP was the respondents’ living arrangement (see Table 2)—interest in PrEP was reported by all GBM living on a military base, compared to approximately 2/3 of GBM living off-base (*p* = 0.016).

**Table 2:** PrEP interest, access and discussion with military healthcare providers (HCP) among GBM active duty servicemembers

	**PrEP Interest (*n* = 93)**		**PrEP Discussion with Military HCP (*n* = 71)**		**PrEP Access (*n* = 45)**	
	Not interested in PrEP (n = 27)	Interested in PrEP (n = 66)	Has not discussed PrEP w/ HCP (n = 32)	Has discussed PrEP w/ HCP (n = 39)	Does not have access to PrEP (n = 13)	Has access to PrEP (n = 32)
**Age** (median; IQR)	30 (26–33)	28 (25–34)	29 (26–35)	27 (25–32)	33 (27–42)	26.5 (24.5–29)^**^
**Race/Ethnicity**						
White/Caucasian	19 (26.8%)	52 (73.2%)	22 (41.5%)	31 (58.5%)	10 (28.6%)	25 (71.4%)
Black/African American	2 (40%)	3 (60%)	2 (50%)	2 (50%)	0 (0%)	1 (100%)
Latinx/Hispanic	3 (33.3%)	6 (66.7%)	4 (44.4%)	5 (55.6%)	0 (0%)	5 (100%)
Asian/Pacific Islander	2 (50%)	2 (50%)	1 (50%)	1 (50%)	1 (50%)	1 (50%)
Multiracial/Other	1 (25%)	3 (75%)	3 (100%)	0 (0%)	2 (100%)	0 (0%)
**Marital Status**						
Single	12 (21.4%)	44 (78.6%)	18 (38.3%)	29 (61.7%)^*^	8 (25%)	24 (75%)
Married/Partnered	14 (43.8%)	18 (56.3%)	13 (68.4%)	6 (31.6%)	5 (55.6%)	4 (44.4%)
Divorced/Separated	1 (20%)	4 (80%)	1 (20%)	4 (80%)	0 (0%)	4 (100%)
**Educational Level**						
High School	1 (16.7%)	5 (83.3%)	3 (60%)	2 (40%)	1 (50%)	1 (50%)
Associates/Some College	5 (29.4%)	12 (70.6%)	8 (53.3%)	7 (46.7%)	2 (18.2%)	9 (81.8%)
Bachelor’s	9 (25.7%)	26 (74.3%)	12 (42.9%)	16 (57.1%)	2 (13.3%)	13 (86.7%)
Graduate School	12 (34.3%)	23 (65.7%)	9 (39.1%)	14 (60.9%)	8 (47.1%)	9 (52.9%)
**Military Branch**						
Air Force	8 (27.6%)	21 (72.4%)	10 (45.5%)	12 (54.5%)	5 (41.7%)	7 (58.3%)
Army	12 (35.3%)	22 (64.7%)	11 (50%)	11 (50%)	5 (33.3%)	10 (66.7%)
Marine Corps	3 (30%)	7 (70%)	2 (25%)	6 (75%)	0 (0%)	6 (100%)
Navy	4 (20%)	16 (80%)	9 (47.4%)	10 (52.6%)	3 (25%)	9 (75%)
**Post**						
On-Base	0 (0%)	12 (100%)^*^	8 (61.5%)	5 (38.5%)	2 (25%)	6 (75%)
Off-Base	27 (33.8%)	53 (66.3%)	23 (40.4%)	34 (59.6%)	11 (29.7%)	26 (70.3%)
**Accessed healthcare in past year**						
No	16 (38.1%)	26 (61.9%)	16 (53.3%)	14 (46.7%)	2 (13.3%)	13 (86.7%)
Yes	11 (21.6%)	40 (78.4%)	16 (39%)	25 (61%)	11 (36.7%)	19 (63.3%)
**Accessed healthcare from military provider in past year**						
No	17 (37%)	29 (63%)	18 (54.5%)	15 (45.5%)	3 (18.8%)	13 (81.3%)
Yes	10 (21.3%)	37 (78.7%)	14 (36.8%)	24 (63.2%)	10 (34.5%)	19 (65.5%)
**Accessed healthcare from civilian provider in past year**						
No	24 (32.4%)	50 (67.6%)	25 (45.5%)	30 (54.5%)	6 (17.6%)	28 (82.4%)^**^
Yes	3 (15.8%)	16 (84.2%)	7 (43.8%)	9 (56.3%)	7 (63.6%)	4 (36.4%)
**Disclosed sexual orientation to healthcare provider**						
No	8 (34.8%)	15 (65.2%)	16 (84.2%)	3 (15.8%)^***^	5 (71.4%)	2 (28.6%)^*^
Yes	18 (26.5%)	50 (73.5%)	16 (31.4%)	35 (68.6%)	8 (21.6%)	29 (78.4%)

*PrEP* HIV pre-exposure prophylaxis; *HCP* healthcare provider

^*^p < .05, ^**^p < .01, ^***^p < .001; Unless otherwise noted, cells represent n(%). Some cells may not equal total amount due to missing data

#### Discussed PrEP with a Military Healthcare Provider

Approximately 59% (*n* = 39/66) of GBM who were interested in PrEP had discussed PrEP with their military doctor. There were statistically significant differences in reports of speaking to a military doctor about PrEP by marital status and sexual orientation disclosure (see Table 2). Greater proportions of GBM who were divorced/separated (*n* = 4, 80%) or single (*n* = 29, 61.7%) had talked to a military doctor about PrEP, compared to GBM who were married/partnered (*n* = 6, 31.6%; *p* = 0.048). Additionally, participants who had disclosed their sexual orientation to their healthcare provider were more likely to have discussed PrEP with a military provider (*n* = 35, 68.6%) compared to those who had not disclosed their sexual orientation to their provider (*n* = 3, 15.8%; *p* < 0.001).

#### PrEP Access

Only about half of the respondents interested in PrEP indicated that they had access to PrEP from their military healthcare provider (*n* = 32/66) (see Table 2). Participants who had access to PrEP tended to be younger (median age 26.5; IQR 24.5–29) compared to those who did not have access to PrEP (median age 33; IQR 27–42) (*p* = 0.009). A higher proportion of participants who disclosed their sexual orientation to a healthcare provider had been able to access PrEP (*n* = 29, 78.4%) compared to those who had not disclosed their sexual orientation to their provider (*n* = 2, 28.6%; *p* = 0.017).

## Discussion

Our study provides insight into the PrEP-related experiences of active duty GBM service members. Quantitative results suggest that, while there is substantial interest in PrEP among GBM service members, key gaps in accessibility remain. Despite 71% of study participants stating interest in PrEP, regardless of age, race/ethnicity, education level, and military service branch, approximately half of those interested in PrEP accessed it within the military health system. Our qualitative data offer a deeper explanation for this discrepancy, suggesting critical barriers at the individual and structural levels. In particular, concerns about gaps in military healthcare providers knowledge about PrEP and negative perceptions about one’s sexual behavior, confidentiality of military medical records and potential unwanted disclosure about sexual orientation and sexual health needs may reduce GBM service members’ willingness to discuss PrEP with their military health provider. Collectively, the qualitative and quantitative results from this study point to a need for system-wide improvement in PrEP accessibility within the military healthcare system.

### Gaps in Knowledge and Skills Among Healthcare Providers

More than half of GBM service members in the quantitative phase of our study with an interest in PrEP also discussed PrEP with their military doctor. However, the participants in our study pointed to their military doctors’ lack of knowledge about PrEP and poor understanding of GBM’s unique health and medical needs. In some cases, participants reported personal or vicarious experiences with military healthcare providers who had no knowledge about PrEP or who expressed biased or discriminatory beliefs toward their GBM patients. Similar encounters by GBM service members have been documented by previous studies conducted with military healthcare providers (Biddix et al., 2013; Blaylock et al., 2018b; Campbell et al., 2017; Goldbach & Castro, 2016). A recent survey conducted among GBM service members reported discomfort in discussing their sex lives with their military healthcare providers, despite indicating their preference to receive PrEP services from the military healthcare system (Gutierrez et al., 2021).

Among 1600 military healthcare providers surveyed between 2015 and 2017, 49% acknowledged that they had poor knowledge about PrEP with only 29% ever prescribing it to their GBM patients. Healthcare providers in this study indicated concerns about PrEP’s adverse effects, adherence challenges, and the need for more evidence of PrEP’s safety and efficacy (Blaylock et al., 2018b). Similarly, Tong et al. (2013), Hakre et al. (2016), Rerucha et al. (2018), and Wilson et al. (2020a) conducted self-administered online surveys of military healthcare providers who admitted to poor knowledge of PrEP; they also reported discomfort in discussing and assessing the health needs of their sexual and gender minority (SGM) patients (Rerucha et al., 2018), prescribing PrEP without clear medical evidence (Wilson et al., 2020b), and offering PrEP to patients who may have low adherence to the medication (Hakre et al., 2016).

It should be noted that some of these studies of military healthcare providers were conducted between 2011 to 2017, during a time when PrEP’s effectiveness in preventing HIV infection had only started to become more widely known in the medical community (Centers for Disease Control & Prevention, 2018). A more recent investigation examining current knowledge, skills and acceptance of PrEP among military health care providers may be warranted. Finally, our findings suggest that while GBM members may be willing to discuss PrEP with their military health care providers, they also acknowledge that substantial gaps in provider knowledge and skills related to PrEP remain. HIV-related training programs for civilian health care providers have been shown to increase providers’ knowledge and familiarity with PrEP, improve confidence in identifying HIV risk, and raise comfort in working with GBM patients (Hart-Cooper et al., 2018; Henny et al., 2019). PrEP detailing interventions, which offer tailored and well-designed PrEP information that are coupled with visits from trained representatives, may be another potential method to improve hesitancy to prescribe PrEP among healthcare providers (Ard et al., 2019). Such programs may be beneficial for military health care providers as well.

### Issues of Confidentiality and Unwanted Sexual Orientation Disclosure

Our qualitative results suggest that active duty GBM may have deep concerns about entrusting their military healthcare providers with their sexual identity and sexual health needs, while our quantitative results indicate that this trust may be necessary in order for GBM service members to disclose their sexual orientation with their healthcare provider, leading to improved PrEP-related communication and uptake. Taken together, our qualitative and quantitative results suggest that a lack of trust among active duty GBM in their military healthcare providers may inhibit open and honest communication about PrEP, which can lead to low PrEP use in this population.

Indeed, disclosure of one’s sexual orientation to a healthcare provider is an important precursor to accessing PrEP, given that the clinical guidelines are population specific, and previous studies report that it is an critical step in PrEP receipt for GBM (Gutierrez et al., 2021; Raifman et al., 2017; Wilson et al., 2020a; Yang et al., 2019). However, among SGM service members, disclosing one’s sexual orientation within the military requires a deep estimation of risks and benefits, with profound and lasting professional and personal implications (McNamara et al., 2020). The fear of being “outed” among LGBT service members may be attributed to the legacy of DADT and other discriminatory policies historically enacted by the DoD (Johnson et al., 2015; McNamara et al., 2020; Ramirez & Sterzing, 2017).

Having to disclose one’s sexual orientation and/or PrEP status to various members of the military due to the lack of confidentiality of military medical records may be a concern among active duty GBM, per our study’s qualitative findings. One study participant, a healthcare provider who had obtained PrEP through the military health care system, described the fear of having his medical records passed around by different providers who had purview to his sexual health needs. This finding is in line with a qualitative study conducted among lesbian military service members who described a strong mistrust of the military healthcare system, noting concerns about the confidentiality of their medical records which discouraged them from accessing military mental health services (Mount et al., 2015).

As our study results suggest, connecting GBM service members with PrEP requires safe and supported disclosure of sexual orientation and/or behavior within the military healthcare system. Previous studies indicate that military healthcare providers may be uncomfortable or hesitant in asking about SGM patients’ sexual orientation and behavior (Biddix et al., 2013; Rerucha et al., 2018). Training military leadership and the military healthcare workforce to build mutual patient-provider trust, foster open dialogue about sex and sexuality, and respond in a supportive and non-judgmental manner to sexual orientation disclosure should be considered as critical components of continuing structural changes to improve the military’s integration of SGM service members (Johnson et al., 2015).

### PrEP Access and Care Barriers Within the Military Healthcare System

A recently issued DoD memorandum (US Department of Defense Agency, 2018) required all military health facilities to offer a “’pathway” to PrEP access through the military health system’s pharmacies and laboratory services, but it did not offer guidance on how to make the navigation to PrEP receipt easier for service members and their beneficiaries. The 2018 DoD memorandum also kept the restrictions in place on limiting PrEP access and availability for service members deployed in certain situations, such as when under hostile-fire or in arduous duty (US Department of Defense Agency, 2018). To date, there is a dearth of published academic and practice articles relating to the implementation of the 2018 DoD directive on PrEP access. Our study findings provide preliminary evidence to suggest that systematic barriers have broadly prevented eligible GBM service members from obtaining PrEP in the military healthcare system, which can help inform appropriate implementation of the 2018 DoD memorandum.

Among the structural barriers described by participants in the current study is the extensive process required to get a PrEP prescription, prompting one participant to access PrEP outside of his military base. Our study found a significant interest in PrEP among those who live on a military base compared to those who lived outside of it, potentially due to reduced opportunities for PrEP access within the military base. Receipt of PrEP-related health services on-base, compared to receiving these health services off-base, was also shown to be preferred in a recent survey of GBM military service members (Gutierrez et al., 2021).

Additionally, prior studies noted a number of administrative and structural issues that limit PrEP accessibility for military service members including governmental budget restrictions, availability of pharmacies and diagnostic services in remote and rural locations, limited PrEP inventory, potential poor adherence to PrEP for military service members with unpredictable training and deployment schedules, and the likely discontinuation of PrEP during deployment, as sexual relations between unmarried service members is commonly viewed as unprofessional in a combat environment (Blaylock et al., 2018b). Furthermore, as noted by members of the Air Force in the current study, limitations on PrEP use have been directed toward military personnel deployed to certain hostile locations as well as pilots and members of the air crew, a restriction that continues to be in place as indicated in the 2018 DoD memorandum (Okulicz et al., 2017; US Department of Defense Agency, 2018).

Overall, our findings point to a significant need for improved and updated policies and training for military leadership and healthcare staff to better support GBM service members’ sexual health and well-being. Mistrust of the military healthcare system, confidentiality concerns, and gaps in healthcare provider knowledge and attitudes about PrEP act as barriers to optimal HIV prevention for GBM active duty military personnel. In past studies, military healthcare providers have called for increased training to better care for their SGM patients, including training on PrEP risk assessment and provision to eligible patients, as there is currently no directive provided by DoD (Blaylock et al., 2018b; Campbell et al., 2017; Hakre et al., 2016; Johnson et al., 2015; Rerucha et al., 2018; Tong et al., 2013; Wilson et al., 2020b). Furthermore, there is a need for strategies to promote the system-wide implementation and evaluation of the 2018 DoD memorandum, to ensure that all military service members have an accessible pathway to PrEP care.

### Peer Networks as a Valuable Asset in PrEP Accessibility

Finally, this study’s qualitative results demonstrate the importance of having a GBM peer network that can offer support as well as act as a resource to exchange valuable health information, including PrEP access and availability. Historically, active duty GBM have always maintained a community built on mutual reliance and support prior to and during DADT (Ramirez & Sterzing, 2017). Similar to the network described by one participant who gained peer support through Facebook, the GBM community during the 1970s developed varied and diverse underground coalitions that allowed them to connect with one another as well as offer financial, legal and emotional support such as when a member was discharged due to the disclosure of his sexual orientation or organize peer-counseling groups for members with HIV diagnoses (Ramirez & Sterzing, 2017). Among non-military GBM, having a more expansive, PrEP aware, and LGBT community connected peer network has shown to improve PrEP uptake, particularly for young GBM and GBM of color (Kuhns et al., 2017; Quinn et al., 2020; Ransome et al., 2019). According to Quinn et al. (2020), peer networks serve to not only provide PrEP information but also to reduce PrEP stigma and increase trust in using PrEP. This was evident in the role of one qualitative participant in our study who, as a healthcare provider taking PrEP himself, fostered trust among his colleagues who were interested in PrEP and shared his expertise in accessing PrEP within the military healthcare system. Future research should aim to better understand the nature and influence of peer networks related to PrEP information sharing and support, and identify strategies for leveraging peer support to improve PrEP access for GBM across the military.

## Limitations

The MAP study, from which data for this analysis were drawn, was one of the first DoD-funded projects aimed specifically at understanding the integration, acceptance, and well-being of LGBT service members. As such, our findings provide key information to address existing gaps in understanding PrEP-related perceptions and experiences among GBM service members and demonstrate the immediate need for a system-wide approach to improve the military healthcare system as it relates to PrEP. However, our study is not without limitations. The cross-sectional nature of the data precluded us from making casual inferences, and the small quantitative sample size of GBM participants prevented multivariable analyses of PrEP interest and accessibility. Additionally, although RDS is a useful strategy for reaching difficult-to-access populations and despite our study team’s best effort to recruit diverse members of active-duty GBM, our sample may not be truly representative of the active duty GBM population.

Moreover, our qualitative sample was limited to participants who spontaneously discussed PrEP as part of larger conversations on health and healthcare, and thus we did not have the opportunity to ask structured questions about PrEP with all participants. Due to this limitation, we were unable to fully document potential community-informed strategies and solutions, such as the leveraging of peer networks, to improve PrEP access among GBM military service members.

Additionally, because our qualitative sample was not linked to our quantitative sample, we were unable to fully combine the experiences and opinions of the qualitative and quantitative samples, nor were we able to directly use the results from the qualitative phase to fully inform the results from the quantitative phase of this study. However, our findings can inform future research which further explores PrEP information, access, and experiences among GBM service members using more detailed qualitative assessment approaches.

Finally, while this study offers a rare glimpse of the PrEP related concerns of active duty GBM military personnel, little is known about PrEP interest and access among specific subgroups of GBM, including Black active duty GBM service members who are at the highest risk for HIV (Okulicz et al., 2017). Our quantitative and qualitative sample predominantly identified as white, and therefore we did not have the ability to identify the PrEP interest and access as well as the facilitators and barriers to PrEP receipt among Black and other GBM service members of color. Additionally, our study did not focus on active-duty heterosexual, lesbian and bisexual females in the military or trans military service members who may also benefit from PrEP. To improve the US Military’s HIV prevention programs, it is imperative to conduct research with larger and more diverse samples of active duty service members.

## Conclusion

The military healthcare system is widely regarded as a premier universal healthcare system that provides free care to its military service members and their beneficiaries. However, this system is failing to sufficiently serve its GBM military personnel, who are at elevated risk for HIV, with regard to PrEP. Issues surrounding confidentiality, gaps in knowledge and mistrust of military healthcare providers, as well as structural barriers to PrEP access limits GBM service members’ ability to prevent HIV acquisition. In fact, such system-wide failures are in direct contrast to the recommendation set forth by the USPSTF recommendations, which not only strongly endorsed PrEP’s efficacy in preventing HIV, but also recommend that PrEP be covered by health insurance programs at no cost particularly for at-risk groups by 2021 (Owens et al., 2019). System-wide programs that build trust in the military healthcare system, build military healthcare providers’ proficiency in caring for its GBM patients, and reduce the bureaucratic and structural barriers to PrEP access must be enacted to support the estimated 12,000 PrEP-eligible service members in the military (Blaylock et al., 2018b).
